# Twenty years of Europol strategic analysis and the harmonisation of EU criminal justice: A meta-analysis

**DOI:** 10.12688/openreseurope.24574.1

**Published:** 2026-08-07

**Authors:** Georgios PAVLIDIS

**Affiliations:** 1 Law, Neapolis University, Paphos, Paphos, Cyprus

**Keywords:** European Union, Europol, criminal law, harmonisation, criminality, organised crime

## Abstract

The objective of this scientific and research article is to conduct a meta-analysis of Europol’s strategic intelligence over the past two decades. The analysis covers all relevant Europol assessments from the 2005 Organised Crime Report to the 2025 Serious and Organised Crime Threat Assessment. The article identifies a progressive transformation, since descriptive reporting has evolved into an intelligence-led and predictive analysis. This progressive transformation reflects a broader institutional shift in EU criminal policy as well. Moreover, the article explores how the successive iterations of Europol’s threat assessments have functioned as soft law instruments and they have supported convergence in enforcement priorities and legal harmonisation. The originality of the article lies in the systematic and longitudinal comparison of relevant Europol reports (2005–2025), including an assessment of their epistemic and policy impact on EU criminal law. The findings of this article contribute to both academic debate and policy-making, because they demonstrate how strategic intelligence can become a driver of harmonisation and governance in the Area of Freedom, Security and Justice.

## 1. Introduction

This article aims to critically examine how Europol’s strategic analyses in the last twenty years, from the 2005 Organised Crime Report ^1^ to the most recent 2025 Serious and Organised Crime Threat Assessment ^2^, have evolved from a descriptive reporting mechanism to an intelligence-led and predictive mechanism, which helps shape EU-wide priorities in law enforcement and criminal policy. Moreover, the article examines whether such non-binding analytical documents, which are produced by an agency lacking legislative competence, have a harmonising effect on EU criminal law. The article advances the hypothesis that Europol’s successive threat assessments have operated as soft law instruments, which influenced policy cycles and priorities at operational level, as well as the common understanding of risk. The article advances a second hypothesis as well, that the methodology and sophistication of Europol’s assessments have established a strong epistemic authority of Europol within the Area of Freedom, Security and Justice.

Existing scholarship has focused primarily on the institutional development of Europol ^3^ and the mechanisms for cross-border police cooperation ^4^. For their part, the epistemic and harmonising functions of Europol’s analytical work remain largely underexplored. Several studies have examined Europol’s accountability and information-exchange frameworks ^5^, while others have analysed the role of Europol in security governance ^6^. Nevertheless, there is a lack of research on the content, methodology and impact of the Organised Crime Report (OCR), Organised Crime Threat Assessment (OCTA) and Serious and Organised Crime Threat Assessment (SOCTA) series over two decades. This article aims to address this gap by applying a meta-analytical method and a longitudinal document analysis. The novelty of this approach lies in considering the strategic assessments of Europol as instruments of epistemic harmonisation. This term describes the process through which shared analytical frameworks support convergence of policies, but without formal legislative approximation. Based on this approach, the article contributes both to the academic literature on EU integration and to the understanding of intelligence-led harmonisation as a tool in the EU fight against crime.

## 2. Research and Results

### 2.1 From reporting to intelligence: the end of the descriptive era

In the early 2000s, the mandate of Europol was focused primarily on information exchange and providing analytical support to Member States to combat serious forms of crime. Europol was established under the 1995 Europol Convention ^7^, based on Article K.3 of the Treaty of Maastricht. Following the adoption of the Lisbon Treaty, legal instruments setting up similar Union entities have taken the form of Council Decisions, and the same approach was applied to Europol ^8^. Currently, the legal basis for Europol is Regulation (EU) 2016/794 ^9^, as amended, most notably by Regulation (EU) 2022/991 ^10^. These Regulations are based on the Treaty on the Functioning of the European Union, specifically Article 88, contrary to previous Council Decisions, which were based on Article 30(1)(b), Article 30(2) and Article 34(2)(c) of the Treaty on European Union.

The powers and tasks of Europol have expanded gradually (processing large datasets, cooperating with private parties, etc.), because the need for coordinated responses to cross-border criminality intensified. This was also necessary due to the enlargement of the EU and the increase of EU Member States from 15 in 1995 to 27 today. In the same period, transnational criminal phenomena, including drug trafficking, human trafficking, and financial crime, have surged due to the opportunities in the borderless internal market ^11^. In this context, the analytical capacity of Europol became an important mechanism for synthesising intelligence gathered from national law enforcement agencies. To produce a consolidated European perspective on organised crime and to map cross-border trends, Europol launched its Organised Crime Report (OCR) series, which sought to provide the Council and Member States with an annual overview of organised criminal groups within the EU, including the structure, typology, and activities of such groups.

The 2005 EU Organised Crime Report ^12^ has been the culmination of this descriptive phase. Being the last report of its kind, it also represented the exhaustion of this phase. OCRs were structured along crime categories rather than strategic priorities. These reports offered a comprehensive, but rather static account of organised criminal activity. The chapters on drug trafficking, trafficking in human beings, financial crime, counterfeiting, and other illicit markets, were complemented by sections on resources, use of violence, and organisational structures. The methodology of the OCR series relied on aggregated national contributions. Neverhtless, these contributions were often uneven in quality and scope, which resulted in documents more diagnostic than predictive. Therefore, the OCR series succeeded in mapping the diversity of organised crime across the Member States, but it fell short of providing forward-looking analysis or actionable intelligence. In other words, it constituted a snapshot of criminal trends, but it lacked analytical depth to guide EU policy or operational responses.

In its foreword, the 2005 report explicitly recognised these limitations and announced an important methodological shift ^13^. This was one of the outcomes in the “The Hague Programme” in the areas of freedom, security and justice, adopted by the European Council in November 2004 ^14^. More specifically, the European Council decided that from 1 January 2006, Europol must replace the crime situation report by annual threat assessments. The objective was to assess serious forms of organised crime, based on information provided by the Member States and input from Eurojust ^15^ and the European Police Chiefs Task Force ^16^. In their turn, these analyses would assist the Council of the EU in establishing strategic priorities and shaping further action. Therefore, the Hague Programme marked the transition from descriptive reports towards more predictive assessments. This reflected a broader shift in EU internal security policy, in accordance with the Hague Programme’s emphasis on intelligence-led policing, as well as on strategic coordination. In this context, the Europol would not serve merely as a repository of information, but it would produce strategic intelligence, valuable both for political decision-making and for setting operational priorities. Therefore, the 2005 OCR must be viewed as threshold: it was the final exercise in descriptive ‘criminography’, before Europol assumed a more central actor in shaping the EU’s intelligence-led approach to organised crime.

### 2.2 The organised crime threat assessments: building an intelligence-led paradigm

The period 2006–2011 is part of an important methodological evolution. The EU Organised Crime Threat Assessment (OCTA) series, which began in 2006, attempted a shift from Europol’s prior descriptive reports towards a threat assessment model that is intelligence-led. The inaugural OCTA introduced a qualitative analysis of organised crime trends, and it drew on existing expertise to anticipate emerging threats ^17^. Early editions of OCTA relied on inputs from multiple sources, such as EU Member States, EU agencies (e.g. Eurojust, Frontex, OLAF, European Monitoring Centre for Drugs and Drug Addiction, currently known as the European Union Drugs Agency) and even third states ^18^. Europol analysts synthesized these sources into a strategic product. Over time, Europol’s methodology became more systematic: by 2008 the OCTA adopted a more structured “conceptual model”, which addressed organised crime groups first, then criminal markets, and finally regional dynamics ^19^. The 2009 OCTA constitutes another milestone, as it provides a unified analysis of group structures, criminal activities and criminal environment into one framework ^20^. The 2009 report did not simply reiterate national findings, but it explicitly focused on identifying complex criminal combinations that were shaping EU-wide organised crime. The scope of the report included a brief survey of all major criminal markets for new trends, a mapping of those trends onto geographic “hubs,” and finally an evaluation of criminal groups’ capabilities. This approach was accompanied by a methodological refinement: Europol amended its procedures in close cooperation with Member States to improve the quality of national contributions year by year. We observe that, by the final OCTA in 2011, the assessment had matured into an even more granular analysis covering three perspectives: criminal commodities, criminal groups, and their geographic areas of operation ^21^. Although the 2011 OCTA’s structure contained detailed thematic sections (drugs, human trafficking, fraud, etc.), each section included cross-cutting analysis of criminal group dynamics and nodal areas for that crime. This differentiates the approach and design of the 2011 report from the 2006 report’s generalized indicators (like use of violence, corruption, or technology). As Europol’s threat assessments became flagship products, they leveraged intelligence from law enforcement databases, private-sector consultations, and even socio-economic trend data. They offered a more nuanced picture of the diversification of organised crime (e.g. into cyber-facilitated fraud and counterfeit goods), as well as its impact on European society.

In this context, it is worth mentioning several important institutional and policy linkages. The OCTA’s development was associated with EU policy initiatives in Justice and Home Affairs. As a direct response to The Hague Programme (2004) ^22^, the OCTA replaced Europol’s old OCR series, marking a change of paradigm, favouring proactive and intelligence-led policing. From the outset, the role of OCTAs was to combine strategic and operational realms, “linking those at the ministerial and political levels with those of practitioners and law enforcement agencies who operate at the front line” ^23^. This was also in line with the European Criminal Intelligence Model (ECIM) ^24^, the EU’s framework for intelligence-led law enforcement. The OCTA did not replace national threat assessments, but it complemented them, since it focused on threats that transcend borders, helping coordinate priorities at regional level. The impact of early OCTAs is evident in the Council Conclusions, which explicitly recognised the need for “intensifying police cooperation, in particular giving an increased role to Europol to support operations” ^25^, shaping EU-wide priorities in subsequent years. Europol’s 2007 and 2008 reports note that these conclusions, drawn from OCTA findings, had significant impact on practice, since they inspired initiatives like the European Police Chiefs Task Force/COSPOL framework, the Baltic Sea Task Force, the Operational Inter-organisational Action Plan to Fight Human Trafficking in Greece (ILAEIRA), and the Maritime analysis and operations centre – narcotics (MAOC-N) in Lisbon ^26^. This confirms that strategic analysis has the potential to inform policy and operations in impactful ways. It is worth noting that in 2010 the Council adopted the EU Policy Cycle for Serious and Organised Crime (EMPACT), as a multi-year planning framework, and it explicitly identified the OCTA as an instrument for EU priority-setting ^27^. Thus, the OCTA series did not simply mirror EU policy evolution, but it actively supported the harmonisation of EU strategies, by providing a shared intelligence picture to guide legislation, funding, and cross-border policing initiatives.

One of the OCTA’s most important analytical innovations was the development of the concept of “criminal hubs” ^28^. This concept refers to major geo-strategic regions where criminal logistics, markets, and groups converge. It marks a departure from nation-bound threat models, and it provides a spatial framework for understanding organised crime at EU level. Early OCTAs acknowledged that such regional contexts matter ^29^. The 2009 OCTA, which formally introduced the hubs concept, attempted to understand organised crime in terms of transnational nodal zones of activity ^30^. It identified five key EU criminal hubs, which exert “wide influence on criminal market dynamics in the EU”. The concept of “criminal hubs” allowed Europol to focus on complex patterns (trafficking flows, convergence points, and facilitating infrastructure) that affect one or more countries. For example, it highlighted how a Balkan/South-East European hub had become a nexus for illicit flows of heroin, cocaine, illegal migrants and trafficking victims into the EU. Subsequent OCTAs refined this framework. The 2011 report reaffirmed the existence of five hubs and underscored shifts within them ^31^. For example, it identified the expansion of the South-East hub to Western Balkans, which together formed a “Balkan axis” of criminal routes into the EU. It also examined how other hubs (e.g. the North-West hub around the Netherlands/Belgium) function as distribution centers for drugs and illicit commodities across Europe. The hub concept allowed the EU to prioritize hotspots of EU-wide significance, guide regional cooperation, and reveal interdependencies (such as feeder zones in Africa or Asia fueling European illicit markets). Comparatively, the 2006 OCTA lacked such spatial analysis, offering only general observations on regional divergences. The hub-based approach illustrates how Europol’s assessments grew more sophisticated in capturing the geography of organised crime, its logistics and threat distribution. It helped ensure that EU-wide priorities (for example, combating West African drug supply chains or Balkan human smuggling networks) were grounded in a spatial intelligence context, to improve the allocation of law enforcement resources to where they would prove most effective.

### 2.3 Strategic and methodological evolution (2013–2025)

The inaugural EU Serious and Organised Crime Threat Assessment (SOCTA) was published in 2013 ^32^. The report adopted an intelligence-led approach to organised crime, identifying threats, as well as new phenomena tied to the internet and the post-2008 economic crisis. Each subsequent edition of SOCTA expanded the scope and depth of Europol’s analysis. By 2017, Europol had undertaken an extensive data collection on EU organised crime, providing a more holistic threat picture. Moreover, the 2017 SOCTA refined further its methodology ^33^. In addition to cataloguing specific crime markets (cybercrime, illicit drugs, human trafficking, migrant smuggling, organised property crime, etc.), the report identified cross-cutting “engines” of criminality. The term refers notably to money laundering, document fraud and online illicit trade, which facilitate most other crimes. The report further also recognized the growing impact of technology, in particular encryption, darknet platforms, and cyber services. It warned that virtually all types of criminal activities were now enabled by digital tools, which become a serious challenge for law enforcement. For its part, the 2021 SOCTA built on this realisation and documented how organised crime networks had adapted to global disruptions (e.g. the COVID-19 pandemic) and to the digitalisation of crime ^34^. According to Europol, cyber-dependent attacks and online-facilitated illicit activities were surging, while certain criminal activities (human trafficking and child sexual exploitation) were moving online almost entirely. For this reason, Europol adopted new analytical models, including scenario planning, to anticipate emerging threats (e.g. the potential impact of a global economic recession). By the 2025 SOCTA, this approach reached full maturity ^35^. The latest report spotlighted how novel crime domains and novel threats, such as environmental crime and AI-driven crime. It described the very “DNA” of organised crime as being transformed by digitalisation. Thus, the editions of SOCTA evolved from a more static model into a more dynamic one, which focuses to new forms of criminality in the digital world.

It is also worth examining how SOCTA integrates with EU frameworks and policy agendas. From the outset, the SOCTA was conceived as an important component of an EU-wide policy cycle. It aimed to ensure that intelligence translated into joint policy priorities. Over time, the SOCTA became part of the EU security frameworks such as EMPACT (the European Multidisciplinary Platform Against Criminal Threats) ^36^ and the Security Union Strategy ^37^. The 2013 report’s findings informed the first EU Policy Cycle (2014–2017) ^38^, and by 2017 the role of Europol in this process was being recognised widely. SOCTA 2017 provided a truly in-depth basis for decision-makers and placed Europol “at the heart of the European security architecture” ^39^. Every four years, SOCTA guides the Council of the EU in setting its multiannual crime priorities. For example, the 2021 SOCTA led to the Council adopting the EU priority crime areas for 2022–2025, from high-risk criminal networks and cyber-attacks to environmental crime, based on Europol’s threat analysis ^40^. The European Commission likewise drew on SOCTA 2021 when formulating the EU Strategy to Tackle Organised Crime 2021–2025 ^41^. In 2021, the EMPACT coordination mechanism, which was originally a time-limited policy cycle, became a permanent instrument, demonstrating the importance of informed priority-setting. The operational mandate of Europol evolved in step with this institutional development. The agency’s founding regulation ^42^ and its 2022 enhancement ^43^ strengthened Europol’s capacity to process big data and partner with private sectors. This reflects a recognition that effective threat assessments require vast information-sharing and analysis. In practice, Europol now serves not just as an “information hub” but as the coordinator of responses to SOCTA-identified threats.

Between 2013 and 2025, the SOCTA series has matured and become an important governance tool for European criminal justice cooperation. Beyond threat identification, it can steer how the EU collectively responds to security challenges. The SOCTA-EMPACT cycle helps harmonise national law enforcement priorities into a unified EU agenda. This addresses the previous lack of coordination, which could be exploited by organised criminals ^44^. Indeed, the implementation of SOCTA’s priorities requires concerted action by Member States, EU agencies, and other stakeholders together. Accordingly, the EMPACT framework covers both preventive and repressive measures, operational and strategic actions, across the Union.

Europol’s 2025 assessment is used by the Council to set common objectives and shape EU initiatives, such as tightening anti-money laundering laws or upgrading cyber-security capabilities in line with identified threats. There is now a new culture of evidence-based policy in EU internal security. In this context, Europol’s analysis lends authority and credibility to EU-wide initiatives, while it also helps align national and EU-level strategies. Therefore, what began as an intelligence product has evolved into an important instrument of governance in the EU fight against serious and organised crime.

### 2.4 Cross-cutting developments in europol’s OCTA/SOCTA (2006–2025)

Europol’s threat assessments over the past two decades reflect changes in crime priorities and narratives. Early OCTA reports concentrated on the “usual suspects”, i.e. traditional high-profile crimes (drug trafficking, financial fraud, human trafficking, etc). They often neglected emerging or “low-profile” threats. However, new crime types gained prominence, as the OCTA series progressed. One of the most interesting examples is environmental crime (e.g. waste trafficking), which evolved from a marginal concern into a recognized threat. Indeed, this type of crime was initially met with reluctance due to limited intelligence, but by the 2010s Europol acknowledged it as a manifestation of organised crime, as a high-profit and low-risk criminal enterprise ^45^. The latest SOCTAs confirms that environmental offenses constitute significant crime threats ^46^. A similar rise is seen in cybercrime, which was once grouped under “technology as facilitator of organise crime” in 2000s OCTAs ^47^. Cybercrime, which includes cyber-dependent attacks and online fraud schemes, has become central in recent SOCTAs, such as SOCTA 2021 ^48^. Meanwhile, human trafficking remains a priority across all editions, but its framing has evolved. Indeed, there is a shift from purely physical exploitation by clandestine networks to a hybrid online-offline criminal enterprise. As shown in recent assessments, human traffickers now recruit and advertise victims almost entirely online, using social media and darknet forums ^49^. In short, crimes like cyber-offenses and environmental crime moved from the periphery of Europol’s reporting to its core, which have been long dominated by drugs and traditional trafficking.

In the period 2006–2025, Europol has refined how it conceptualises organised crime groups (OCG) and how it incorporates geographic analysis into its strategy. For example, the 2009 OCTA used an OCG typology based on each group’s strategic center of interest and use of intimidation or corruption ^50^. There was a classification of groups based on their propensity for violence against society, corruption of law enforcement, etc. This can be explained by the early focus on relatively structured mafias, which were territorially grounded. However, Europol’s view of OCGs expanded over time into a more fluid network model. By the 2010s it was evident that OCG structures vary widely from strong hierarchical groups to flexible entrepreneurial rings. The SOCTA 2021 went so far as to note that many criminal networks mirror legitimate corporations and their components (managerial hierarchies, specialist service providers, adaptable business models, etc.) ^51^. The recent assessments by Europol portray cooperation among different OCGs as fluid, systematic, and profit-oriented, not limited by rigid ethnic or territorial boundaries that earlier assessments often emphasized ^52^. Regarding the use of geographic intelligence to map crime, the 2011 OCTA delineated key “criminal hubs”, as already mentioned. Subsequent editions built on this spatial model. The SOCTA reports in the 2020s trace the evolution of transnational trafficking routes; they also demonstrate the proliferation of new convergence zones where routes intersect. Moreover, Europol’s 2025 assessment recognizes that nearby regions can incubate criminal threats “due to their proximity and the borderless nature of serious and organised crime” ^53^. For this reason, it stresses monitoring beyond the EU’s borders.

Finally, an important theme across OCTA/SOCTA editions has been the impact of digital technology on the organised crime landscape. By 2011, Europol already observed that “Internet technology has … emerged as a key facilitator for the vast majority of offline organised crime”, enabling everything from illicit trafficking to fraud and money laundering across borders ^54^. This trend has accelerated since then. The 2025 SOCTA concludes that virtually all serious criminal activities now have an online component, since criminals exploit encrypted communications, darknet platforms, and cryptocurrency to obscure their operations and coordinate across borders ^55^. New technologies are being weaponised, especially artificial intelligence which is cited as “dramatically enhancing” criminal capabilities and scaling up cyber-fraud, ransomware, and online child exploitation schemes. Importantly, the impact of technology impact is twofold: it changes how criminals commit crimes, but it also guides how law enforcement must fight them. Following this logic, the methodology of Europol has become more data-driven and more anticipatory. In the context of modern SOCTAs, a mixed-method intelligence approach is adopted, using internal databases, Member State contributions, and vetted open-source information to generate a holistic analysis. As already mentioned, the emphasis is on proactive and “intelligence-led” policing. In this context, digitalisation has become a defining factor in both contemporary organised crime and Europe’s efforts to counter it.

### 2.5 Europol’s threat assessments and harmonisation of EU criminal justice

In this section of our research, we examine how Europol’s threat assessments have contributed to the harmonisation of EU criminal justice. We have identified three dimensions: a) the normative influence of Europol’s OCTA/SOCTA reports on EU law and policy; b) the use of Europol’s OCTA/SOCTA reports as soft-law tool for operational harmonisation; c) the rise of Europol as epistemic authority, a knowledge authority whose analyses frame Member States’ perceptions of threats and best practices.

Regarding the first dimension, Europol’s threat assessments have exerted significant normative influence on EU policymaking in the field of criminal justice. ^56^ From the first OCTA in 2006 through the SOCTAs up to 2025, Europol’s assessments have set the agenda and spotlighted urgent crime threats. The Justice and Home Affairs Council, via its Conclusions, has explicitly used OCTA/SOCTA findings to define the EU’s priorities for combating serious crime. For example, the OCTA 2013 provided the basis for the Council to adopt priority crime areas for 2011–2013, which then guided EU initiatives in those fields ^57^. This practice became part of the EU Policy Cycle, since each four-year SOCTA informs the Council’s choice of priority crime threats to tackle at EU level. For its part, the European Commission aligns its internal security strategies and legislative proposals with these priorities. This was the case of the 2021–2025 EU Strategy to Tackle Organised Crime ^58^, which drew on Europol’s SOCTA analysis. Europol’s threat assessments have also facilitated the adoption of new or updated legal instruments. For instance, the emergence of environmental crime as a threat in Europol’s analysis led to renewed EU legislative efforts in that domain. Europol’s analysis has also informed other legislative initiatives, such as the Asset Recovery and Confiscation Directive ^59^, the recently adopted 6 ^th^ AML/CFT Directive ^60^ and the new AML/CFT Regulation ^61^. Thus, while not legally binding, OCTA/SOCTA reports have normatively steered EU policies toward a harmonised set of priorities.

Regarding the second dimension, Europol’s OCTA and SOCTA also function as soft law instruments. ^62^ Although they lack formal coercive force, they help harmonise operational practice, since the assessments initiate a cycle of coordination. In this cycle, the assessments are the first step of each EMPACT, through which the priority areas lead to joint operational efforts by Member States, Europol, and other agencies. Notably, the priorities identified by the threat assessments are implemented via Multi-Annual Strategic Plans and yearly Operational Action Plans, which are jointly drafted and monitored by Member States under the guidance of Europol and the Council’s Standing Committee on Operational Cooperation on Internal Security (COSI) ^63^. The SOCTA recommendations are not hard law, but they are part of a cooperative framework that aligns the activities of national authorities. Since the introduction of this mechanism, Europol has observed a convergence in law enforcement operations: the number and effectiveness of cross-border investigations, information sharing, and joint interventions have improved in the designated priority areas. For instance, after setting West African drug trafficking as a priority, several coordinated projects pooled resources from multiple countries to disrupt those networks ^64^. Similar initiatives illustrate how a common EU threat picture can lead to more effective action on the ground. Furthermore, Europol’s role in hosting analytical platforms and secure channels (SIENA) allows operational intelligence to be shared and fed back into the knowledge cycle. In effect, the OCTA/SOCTA reports act as a soft-law blueprint for cooperation, since they can standardise objectives and procedures (e.g. by promoting best practices and joint training).

Finally, it can be argued that the evolution of Europol’s threat assessments has established Europol as an epistemic authority in EU criminal justice. Europol is recognised as a central source of expert knowledge that shapes how all Member States perceive and respond to criminal threats. The SOCTA reports are based on systematic analysis of data contributed by all Member States. They pool thousands of law enforcement intelligence inputs into one assessment. Thus, they create a single EU narrative about threats (“the most comprehensive, forward-looking analysis to date”) ^65^. For their part, national authorities rely increasingly on this collective narrative when setting their own priorities. They also share and use the concepts and terminology developed by Europol in a uniform way. Moreover, the SOCTA/EMPACT cycle, disseminates best practices and effective strategies against the identified threats, further influencing the tactical choices at the level of the Member States. In academic terms, Europol has become an “epistemic community” leader ^66^, since it gathers and validates knowledge and legitimates threat perceptions. It can be argued that this has led to a form of “cognitive harmonisation ^”^, since national law enforcement and policymakers speak the same language and agree on what works to combat organized crime threats. Importantly, when all actors and stakeholders acknowledge the same threats and reference a common knowledge base, there are more chances for coordinated solutions.

## Discussion and conclusions

The aim of this article was to examine how Europol’s non-binding strategic threat assessments have evolved and shaped EU policies. The research confirms the hypothesis that Europol’s OCTA and SOCTA reports have evolved beyond descriptive intelligence to become instruments of soft law and strategic governance. Over two decades, they have supported operational convergence (through EMPACT), epistemic consolidation (through the creation of shared threat perceptions and analytical language), and normative influence (by guiding legislative and policy priorities). The findings of this article support the notion that harmonisation in EU criminal law may arise not only through formal legislative procedures but also through coordination processes initiated by agencies like Europol.

The originality of this study lies in its longitudinal meta-analysis of Europol’s flagship reports from 2005 to 2025. The study examines the structural evolution of these reports, as well as the issues of policy integration and conceptual maturation. It finds evidence of a certain “epistemic harmonisation,” whereby shared threat assessments generate convergence in EU and national priorities. This idea is underexplored in the literature, which tends to focus on institutional design and judicial cooperation. In contrast, this article explores the role of Europol analysis in constructing common security imaginaries. Thus, it extends prior work on the operational development of Europol; it builds on theories of epistemic governance ^67^, since it illustrates how the intelligence outputs can set the agenda and diffuse norms.

These findings have legal and institutional implications. From a legal perspective, they demonstrate the need to account for soft law tools in the process of EU criminal law harmonisation ^68^. Indeed, Europol’s analyses have informed Council Conclusions, Commission strategies, and legislative initiatives. From an institutional perspective, we argue that Europol emerges as an epistemic authority. This reconfigures the balance of knowledge-power within EU internal security. Of course, this raises new questions, such as the methodological robustness, transparency, and democratic accountability of this process ^69^. Future research could further explore how other agencies (e.g. Frontex, Eurojust) contribute to or challenge the epistemic centrality of Europol. Ultimately, the findings of this study reaffirm the importance of strategic intelligence, which has the potential to shape major policy choices in the field EU criminal justice.

## Data availability statement

No new datasets were generated in the course of this research. The study is based exclusively on the analysis of publicly available sources, including Europol reports, EU legal instruments, and published academic literature.

## Ethics and consent statement

Ethical approval and consent were not required.

## Footnotes

^1^
Europol, *OCR 2005: EU Organised Crime Report - Public version*, The Hague 2005.

^2^
Europol, *SOCTA 2025: EU Serious and Organised Crime Threat Assessment: The Changing DNA of Serious and Organised Crime*, Luxembourg 2025.

^3^
Busuioc M., Groenleer M., *Beyond Design: The Evolution of Europol and Eurojust*, [in:] *Justice and Home Affairs Agencies in the European Union*, Routledge 2016, pp. 13–32.

^4^
Fägersten B., *Bureaucratic resistance to international intelligence cooperation–the case of Europol*, “Intelligence and National Security” 2010, vol. 25(4), pp. 500–520; Safjanski T., *Barriers to the operational effectiveness of Europol*, “Internal Security” 2013, vol. 5(1), p. 53 ff.

^5^
Cordeel G., *Europe’s police information exchange: an exercise in information management*, “Journal of Police Studies/Cahiers Politiestudies” 2010, vol. 3(16), pp. 107–119; Fiodorova A., *Information exchange and EU law enforcement*, Routledge 2018.

^6^
Riekmann S., *Security, freedom and accountability: Europol and Frontex,* [in:] *Security versus Justice?* Routledge 2016, pp. 19–34.

^7^
Convention based on Article K.3 of the Treaty on European Union, on the establishment of a European Police Office - Europol Convention (OJ C 316, 27.11.1995, pp. 2–32).

^8^
Council Decision of 6 April 2009 establishing the European Police Office – Europol (OJ L 121, 15.5.2009, pp. 37–66); see also De Moor A., Vermeulen G., *The Europol council decision: transforming Europol into an agency of the European Union*, “Common Market Law Review” 2010 vol. 47(4), pp. 1089–1121.

^9^
Regulation (EU) 2016/794 of the European Parliament and of the Council of 11 May 2016 on the European Union Agency for Law Enforcement Cooperation (Europol) and replacing and repealing Council Decisions 2009/371/JHA, 2009/934/JHA, 2009/935/JHA, 2009/936/JHA and 2009/968/JHA (OJ L 135, 24.5.2016, pp. 53–114).

^10^
Regulation (EU) 2022/991 of the European Parliament and of the Council of 8 June 2022 amending Regulation (EU) 2016/794, as regards Europol’s cooperation with private parties, the processing of personal data by Europol in support of criminal investigations, and Europol’s role in research and innovation (OJ L 169, 27.6.2022, pp. 1–42).

^11^
Herlin-Karnell E., *EU criminal law and its complicated relationship to the internal market*, [in:] *Research Handbook on EU Criminal Law*, Edward Elgar Publishing 2024, pp. 252–264.

^12^
OCR 2005, *op. cit.*

^13^
More specifically, “the change from ‘report’ to ‘threat assessment’ indicates the major challenge for the new document to be produced: to move away from a more descriptive report towards a more far-reaching predictive assessment, which will allow for a forward-looking strategic and, in a second step, operational priority setting”.

^14^
The Hague Programme: strengthening freedom, security and justice in the European Union (OJ C 53, 3.3.2005, pp. 1–14).

^15^
This is the European Union Agency for Criminal Justice Cooperation (Eurojust), which operates on the basis of Article 85 of the Treaty on the Functioning of the European Union, as well as Regulation (EU) 2018/1727 of the European Parliament and of the Council of 14 November 2018 on the European Union Agency for Criminal Justice Cooperation (Eurojust), and replacing and repealing Council Decision 2002/187/JHA (OJ L 295, 21.11.2018, pp. 138–183).

^16^
See Tampere European Council, 15 and 16 October 1999, Presidency Conclusions, SN 200/99, par. 44.

^17^
Europol, *OCTA 2006: EU Organised Crime Threat Assessment*, The Hague 2006, p. 23.

^18^
Europol, *OCTA 2007: EU Organised Crime Threat Assessment*, The Hague 2007, p. 5.

^19^
Europol, *OCTA 2008: EU Organised Crime Threat Assessment*, The Hague 2008, p. 8.

^20^
Europol, *OCTA 2009: EU Organised Crime Threat Assessment*, The Hague 2009, p. 9.

^21^
Europol, *OCTA 2011: EU Organised Crime Threat Assessment*, The Hague 2011.

^22^
Balzacq T., Carrera S., *The Hague programme: The long road to freedom, security and justice*, [in:] *Security Versus Freedom?*, Routledge 2016, pp. 1–32; The Hague Programme: strengthening freedom, security and justice in the European Union (OJ C 53, 3.3.2005, pp. 1–14).

^23^
OCTA 2006, *op. cit.*, foreword, p. 3.

^24^
Gruszczak A., *The EU criminal intelligence model: problems and issues*, [in:] *EU Criminal Law and Policy*, Routledge 2016, pp. 149–167.

^25^
Council of the European Union, Presidency Conclusions, Document No 10633/1/06, Brussels, 17 July 2006, par. 6.

^26^
OCTA 2007, *op. cit.*, p. 5; OCTA 2008, *op. cit.*, p. 5.

^27^
Council of the European Union, Council Conclusions, Document No 15358/10: The Creation and Implementation of a EU Policy Cycle for Organised Crime and Serious International Crime, Brussels, 8–9 November 2010. The initial, reduced, EU Policy Cycle for organised and serious international crime was implemented between 2012–2013. This was followed by two fully-fledged cycles between 2014–2017 and 2018–2021. In 2021, the Council of the European Union decided on the permanent continuation of the EU Policy Cycle for organised and serious international crime, while it also introduced the change of name to ‘EMPACT’. The four-year periodicity of its steps remained unchanged. The current EMPACT 2022+ covers the period 2022–2025.

^28^
Keefe P., *The geography of badness: Mapping the hubs of the illicit global economy*, [in:] *Convergence: Illicit networks and national security in the age of globalization,* National Defense University Press 2013, pp. 97–110.

^29^
OCTA 2007, *op. cit.*, p. 5; OCTA 2008, *op. cit.*, p. 7.

^30^
OCTA 2009, *op. cit.*, p. 13.

^31^
OCTA 2011, *op. cit.*, p. 50 (Annex).

^32^
Europol, *SOCTA 2013: EU Serious and Organised Crime Threat Assessment*, The Hague 2013.

^33^
Europol, *SOCTA 2017: EU Serious and Organised Crime Threat Assessment*, The Hague 2017, p. 9.

^34^
Europol, *SOCTA 2021: EU Serious and Organised Crime Threat Assessment: A Corrupting Influence: The Infiltration and Undermining of Europe’s Economy and Society by Organised Crime*, Luxembourg 2021, pp. 90–97.

^35^
SOCTA 2025, *op. cit.*

^36^
See e.g. Council of the European Union, Council Conclusions on the permanent continuation of the EU Policy Cycle for organised and serious international crime: EMPACT 2022 +, Document No 6481/21, Brussels, 26 February 2021.

^37^
Communication from the Commission to the European Parliament, the European Council, the Council, the European Economic and Social Committee and the Committee of the Regions on the EU Security Union Strategy (COM/2020/605).

^38^
Council of the European Union, The EU Policy Cycle to Tackle Organised and Serious International Crime, Document No QC-01-14-638, Brussels 2014.

^39^
SOCTA 2017, *op. cit.*, p. 8.

^40^
Council of the European Union, Council Conclusions setting the EU’s priorities for the fight against serious and organised crime for EMPACT 2022–2025, Document No 8665/21, Brussels, 12 May 2021.

^41^
Communication from the Commission to the European Parliament, the Council, the European Economic and Social Committee and the Committee of the Regions on the EU Strategy to tackle Organised Crime 2021–2025 (COM/2021/170).

^42^
Regulation (EU) 2016/794, *op. cit.*

^43^
Regulation (EU) 2022/991 *op. cit.*

^44^
Block L., *Combating organized crime in Europe: practicalities of police cooperation*, “Policing: A Journal of Policy and Practice” 2008, vol. 2(1), pp. 74–81.

^45^
OCTA 2011, *op. cit.*, p. 38.

^46^
SOCTA 2025, *op. cit.*, p. 64.

^47^
OCTA 2006, *op. cit.*, p. 18.

^48^
SOCTA 2021, *op. cit.*, pp. 38–44.

^49^
SOCTA 2025, *op. cit.*, p. 48.

^50^
OCTA 2009, *op. cit.*, p. 17 ff.

^51^
SOCTA 2021, *op. cit.*, pp. 19–25.

^52^
SOCTA 2021, *op. cit.*, p. 10.

^53^
SOCTA 2025, *op. cit.*, p. 84.

^54^
OCTA 2011, *op. cit.*, p. 9.

^55^
SOCTA 2025, *op. cit.*, pp. 88–89.

^56^
Carrapiço H., Trauner F., *Europol and its influence on EU policy-making on organized crime: Analyzing governance dynamics and opportunities*, [in:] *Justice and Home Affairs Agencies in the European Union*, Routledge 2016, pp. 85–99.

^57^
Council of the European Union, Council Conclusions on setting the EU’s priorities for the fight against serious and organised crime between 2014 and 2017, Document No 12095/13, Luxembourg, 6–7 June 2013.

^58^
The EU Strategy To Tackle Organised Crime 2021–2025, *op. cit.*, section 1.2.

^59^
Pavlidis G., *Confiscation internationale: instruments internationaux, droit de l’Union européenne, droit suisse*, Zurich: Schulthess 2012; Pavlidis G., *Asset recovery in the European Union: implementing a “no safe haven” strategy for illicit proceeds*, “Journal of Money Laundering Control” 2022, vol. 25(1), pp. 109–117; Pavlidis G., *Transforming asset recovery offices (AROs) to enhance their role in combating money laundering*, “Journal of Money Laundering Control” 2025, vol. 28(2), pp. 327–340.

^60^
Directive (EU) 2024/1640 of the European Parliament and of the Council of 31 May 2024 on the mechanisms to be put in place by Member States for the prevention of the use of the financial system for the purposes of money laundering or terrorist financing, amending Directive(EU) 2019/1937, and amending and repealing Directive (EU) 2015/849 (OJ L, 2024/1640, 19.6.2024).

^61^
Regulation (EU) 2024/1624 of the European Parliament and of the Council of 31 May 2024 on the prevention of the use of the financial system for the purposes of money laundering or terrorist financing (OJ L, 2024/1624, 19.6.2024).

^62^
Ausfelder A. *et al.*, *EU soft-law: Non-binding but enforceable*, “European Law Journal” 2024, vol. 30(4), pp. 668–684.

^63^
See e.g. Europol Programming Document 2024–2026, adopted by the Management Board of Europol on 28 November 2023, p. 5.

^64^
See for example the project Organised Crime: West African Response to Trafficking (OCWAR–T), funded by the European Union and the German Federal Foreign Office (GFFO).

^65^
SOCTA 2025, *op. cit.*, Executive Summary.

^66^
Miller H., Fox C., *The epistemic community*, “Administration & Society” 2001, vol. 32(6), pp. 668–685; Verdun A., *The role of the Delors Committee in the creation of EMU: an epistemic community?*, “Journal of European public policy” 1999 vol. 6(2), pp. 308–328.

^67^
Alasuutari P., Qadir A., *Epistemic governance: An approach to the politics of policy-making *, “European Journal of Cultural and Political Sociology” 2014 1(1), pp. 67–84; Raman S., *Science, uncertainty and the normative question of epistemic governance in policymaking*, [in:] *Knowledge, Technology and Law*, Routledge 2014, pp. 17–32.

^68^
Terpan F., *Soft Law in the European Union—The Changing Nature of EU Law*, “European Law Journal” 2015 21(1), pp. 68–96; Volpato A., *The legal effects of harmonised standards in EU law: From hard to soft law, and back?* [in:] *The Legal Effects of EU Soft Law*, Edward Elgar Publishing 2023, pp. 193–212.

^69^
Sheptycki J., *To go beyond the cycle of intelligence-led policing*, [in:] *Understanding the intelligence cycle*, Routledge 2013, pp. 99–118.

