# Supplementary material for: Twenty years of Europol strategic analysis and the harmonisation of EU criminal justice: A meta-analysis
Source: Open Res Eur. 2026 Aug 7;6:279. [Version 1] doi: 10.12688/openreseurope.24574.1 (PMC13560729; doi:10.12688/openreseurope.24574.1)
Supplement: Peer review history

# Reviewer report: Reviewer response for version 1

**DOI:** 10.21956/openreseurope.26625.r78917

**Published:** 2026-08-22

**Authors:** Tomasz Michał Matras

**Affiliations:** 1 Institute of International and Security Studies, University of Wrocław, Wrocław, Poland

The article entitled „Twenty years of Europol strategic analysis and the harmonisation of EU criminal justice: A meta-analysis” analyses the evolution of Europol’s strategic assessments between 2005 and 2025 and their significance for harmonisation processes in the field of EU criminal policy and criminal justice. The author examines successive types of reports – from the *Organised Crime Report* (OCR), through the *Organised Crime Threat Assessment* (OCTA), to the *Serious and Organised Crime Threat Assessment* (SOCTA) – focusing on changes in their methodology, thematic scope, and institutional significance. In addition to the Introduction and the *Discussion and Conclusions* section, the article contains a *Research and Results* section divided into five subsections, which in practice form two main lines of argument. The first presents the evolution of the reports from primarily descriptive documents towards analyses increasingly based on risk assessment and the identification of future threats.

 The second focuses on their broader significance, including the normative dimension relating to EU policy and law, the role of the reports in “soft” operational harmonisation, and the growing importance of Europol as an epistemic authority. The article is written in a clear and accessible manner, and its structure is logical. The argument develops consistently from a reconstruction of the changes occurring in Europol’s reports to an assessment of their broader significance for EU security policy, allowing the reader to follow the author’s reasoning.

 The topic is appropriately situated within the context of the existing literature, although this is not the first study to address Europol’s reports. Previous literature has already examined, among other issues, the methodological foundations of OCTA and the way in which organised crime and threat are defined. Nevertheless, the added value of the article lies in bringing together successive stages in the evolution of Europol’s reports within a single longitudinal perspective covering the period 2005–2025. The author does not limit the analysis to the assessment of a single report or one methodological change, but reconstructs the long-term transformation of Europol’s strategic analyses and links it to broader institutional and normative consequences for the coordination of EU security policy. Particularly valuable is the demonstration that harmonisation can also be initiated or reinforced by institutions that formally have no legislative powers. Europol does not make law, but by identifying and prioritising particular threats it can influence their presence in the EU’s institutional and political discourse and inspire institutional and legislative action. In this context, the two research hypotheses formulated in the introduction are appropriate, as they concern the impact of Europol’s reports beyond their original analytical function. From a substantive perspective, the analysis correctly presents the evolution of the methodology and character of the reports – from the more descriptive OCR, through the threat assessment-based OCTA, to the increasingly strategic and forward-looking SOCTA. Another strength of the article is that the analysis is not limited to a simple comparison of the three types of reports, but also shows changes occurring within successive editions of OCTA and SOCTA. The evolution of the range of threats identified by Europol is also presented effectively.

 The author shows how, over the course of two decades, the scope of the analyses expanded from more traditional forms of organised crime to issues related, among others, to darknet platforms, cybercrime services, environmental crime, and the use of artificial intelligence. The article also convincingly presents examples of how Europol’s analyses translated into institutional and operational initiatives, including the European Police Chiefs Task Force/COSPOL, the Baltic Sea Task Force, and MAOC-N. These examples reinforce the argument that Europol’s reports do not remain merely analytical documents, but may influence the practice of police cooperation and strengthen Europol’s position within the European security architecture. At the same time, two elements of the text could be clarified somewhat further. First, it would be useful to state explicitly in the introduction how many reports were included in the analysis. The individual documents are discussed later in the article, but the reader is not provided at the outset with a clear indication of the size and composition of the research sample. Second, the author accurately describes the transition from OCTA to SOCTA and links SOCTA to the *EU Policy Cycle*, but does not sufficiently explain the change in the frequency with which the reports were published. OCTA was initially prepared on an essentially annual basis, whereas full SOCTA editions have, since 2013, generally been published at four-year intervals.

 This change was not merely a modification of Europol’s publication practice but was connected with the institutionalisation of the multiannual *EU Policy Cycle*. The conclusions presented in the article are generally appropriate and consistent both with the analysis conducted and with the existing literature. Particularly convincing is the argument that Europol’s significance should not be assessed solely through the prism of the formal scope of its competences. Over the twenty-year period under analysis, the Agency increasingly positioned itself at the centre of the European security architecture, while its analyses became an important source of knowledge used in defining threats and setting operational priorities. On this basis, the author reasonably points to Europol’s growing importance as an epistemic authority and to the possibility that its reports may exert an indirect harmonising influence, despite Europol’s lack of formal legislative powers.

Are all factual statements correct and adequately supported by citations?

Yes

Is the review written in accessible language?

Yes

Are the conclusions drawn appropriate in the context of the current research literature?

Yes

Is the topic of the review discussed comprehensively in the context of the current literature?

Yes

Reviewer Expertise:

Anti-Money Laundering (AML) policy, financial crime, tax optimisation beneficial ownership transparency, European Union regulatory and security policy

I confirm that I have read this submission and believe that I have an appropriate level of expertise to confirm that it is of an acceptable scientific standard.

# Reviewer report: Reviewer response for version 1

**DOI:** 10.21956/openreseurope.26625.r78915

**Published:** 2026-08-19

**Authors:** Todor Kolarov

**Affiliations:** 1 New Bulgarian University, Sofia, Bulgaria

The article “Twenty Years of Europol Strategic Analysis and the Harmonization of EU Criminal Justice: A Meta-Analysis” consists of three parts – Introduction, Research and Results, and Discussion and Conclusions. The piece under review has a clear structure. In Section 2, Research and Results, it outlines three main periods in the evolution of the Europol EU crime assessment through SOCTA report and its predecessor (subsections 2.1 to 2.3). Further, it emphasizes their importance for the holistic view to the crime situation in the EU and identification of backstage criminal nexus (subsection 2.4). Finally, it analyzes the impact of the evolving treat assessment report in three distinct functional aspects, namely the normative influence of SOCTA reports on EU law and policy, the use of SOCTA reports as soft-law tool, and the rise of Europol as an epistemic authority (subsection 2.5).

 The goal of the article is clearly set – to demonstrate the role of strategic intelligence as a driver of harmonization and governance in the area of Freedom, Security and Justice. A noteworthy mention with this respect is the discussion in the article of the interplay between EMPACT and SOCTA, in light of the strategic importance of the EU Policy Cycle. The research methods are plainly identified by the author in the introduction, namely “meta-analytical method and a longitudinal document analysis”.

 The multitude of resources used support the findings of the article and confirm that Prof. Pavlidis performed a thorough preliminary academic research. There are 69 references, 16 of which are citations of various authors, including 1 self-citation.

 Although the following does not affect the positive assessment of the article, an alternative structural approach may delineate the historical review of the evolution of the SOCTA reports in subsections 2.1 to 2.3 in a separate section of the article from the more analytical subsections 2.4 and 2.5. The latter two may be in a separate section.

 In light of the above, I submit a positive peer-review assessment of the article and support its indexing. I assess the article as **APPROVED**.

Are all factual statements correct and adequately supported by citations?

Yes

Is the review written in accessible language?

Yes

Are the conclusions drawn appropriate in the context of the current research literature?

Yes

Is the topic of the review discussed comprehensively in the context of the current literature?

Yes

Reviewer Expertise:

EU Law, Asset Recovery, Anti-Money Laundering, etc.

I confirm that I have read this submission and believe that I have an appropriate level of expertise to confirm that it is of an acceptable scientific standard.

# Reviewer report: Reviewer response for version 1

**DOI:** 10.21956/openreseurope.26625.r78918

**Published:** 2026-08-18

**Authors:** Marek Fryšták

**Affiliations:** 1 Masarykova univerzita, Brno, Czech Republic

The author has chosen a highly topical subject that has not yet been the subject of scholarly discussion, and no one has addressed it in the same context or to the same extent as the author. It is therefore a completely innovative topic, though it naturally draws on a sufficient body of literature, even if that literature addresses the subject matter in different contexts.  The author’s in-depth analysis and the conclusions drawn from it are valuable for theory and, in particular, for practical application, even though they could have been more extensive; nevertheless, they can at least serve as a springboard for further reflection.

Are all factual statements correct and adequately supported by citations?

Yes

Is the review written in accessible language?

Yes

Are the conclusions drawn appropriate in the context of the current research literature?

Partly

Is the topic of the review discussed comprehensively in the context of the current literature?

Partly

Reviewer Expertise:

criminal law, criminalistics, investigation, organize crime

I confirm that I have read this submission and believe that I have an appropriate level of expertise to confirm that it is of an acceptable scientific standard.
